# Zinc Protects Articular Chondrocytes through Changes in Nrf2-Mediated Antioxidants, Cytokines and Matrix Metalloproteinases

**DOI:** 10.3390/nu10040471

**Published:** 2018-04-11

**Authors:** Tzu-Ching Huang, Wen-Tsan Chang, Yu-Chen Hu, Bau-Shan Hsieh, Hsiao-Ling Cheng, Jeng-Hsien Yen, Pu-Rong Chiu, Kee-Lung Chang

**Affiliations:** 1 Graduate Institute of Medicine, College of Medicine, Kaohsiung Medical University, Kaohsiung 80708, Taiwan; huangtavia@gmail.com (T.-C.H.); jehsye@kmu.edu.tw (J.-H.Y.); 2 Department of Biochemistry, School of Medicine, College of Medicine, Kaohsiung Medical University, Kaohsiung 80708, Taiwan; chingshouhu@gmail.com (Y.-C.H.); hsiehbs@gmail.com (B.-S.H.); chenghl.tanya@gmail.com (H.-L.C.); 3 Department of Surgery, School of Medicine, College of Medicine, Kaohsiung Medical University, Kaohsiung 80708, Taiwan; wtchang@kmu.edu.tw; 4 Division of General and Digestive and Pancreatic Surgery, Department of Surgery, Kaohsiung Medical University Hospital, Kaohsiung Medical University, Kaohsiung 80756, Taiwan; 5 Institute of Medical Science and Technology, College of Sciences, National Sun Yat-sen University, Kaohsiung 80424, Taiwan; 6 Department of Medical Research, Kaohsiung Medical University Hospital, Kaohsiung Medical University, Kaohsiung 80756, Taiwan

**Keywords:** osteoarthritis, zinc, antioxidant, cytokine, matrix metalloproteinase

## Abstract

Osteoarthritis (OA) is an age-related degenerative joint disease characterized by high oxidative stress, chondrocyte death and cartilage damage. Zinc has been implicated in the antioxidant capacity of the cell, and its deficiency might inhibit chondrocyte proliferation. The present study examined the potential of zinc as a preventive supplement against OA using the in vitro chondrosarcoma cell line SW1353 and an in vivo Wistar rat model to mimic OA progress induced by monosodium iodoacetate (MIA). The results demonstrated that, in SW1353 cells, 5 μM MIA exposure increased oxidative stress and decreased the expression of GPx1 and Mn-SOD but still increased GSH levels and HO-1 expression and enhanced the expression of interleukin (IL)-10, IL-1β, and matrix metalloproteinase (MMP)-13. Zinc addition could block these changes. Besides, the expression of Nrf2 and phosphorylated (p)-Akt was dramatically increased, implicating the p-Akt/Nrf2 pathway in the effects of zinc on MIA-treated cells. A rat model achieved similar results as those of cell culture, and 1.6 mg/kg/day of zinc supplementation is sufficient to prevent OA progress, while 8.0 mg/kg/day of zinc supplementation does not have a better effect. These findings indicate that zinc supplementation exerts a preventive effect with respect to MIA-induced OA progress.

## 1. Introduction

Osteoarthritis (OA) is a common degenerative joint disease in the knees, hips, spine, and fingers that is characterized by the progressive abrasion of articular cartilage and remodeling of the underlying bone in the synovial joints, potentially resulting in disability in the aging population [1]. The cartilage of OA patients expresses high IL-1 levels, causing arthritic inflammation and cartilage/bone destruction. Moreover, elevated IL-1 levels induce extracellular matrix (ECM) and matrix metalloproteinase (MMP) expression in chondrocytes [2,3]. In addition, reactive oxygen species (ROS) augment OA [4]. Age-related oxidative stress makes chondrocytes more susceptible to death in humans [5] and rats [6]. Nuclear factor erythroid 2-related factor (Nrf2) activates antioxidative capacities to maintain the integrity of cells and provide protection against oxidative stress [7]. These activated defense systems include superoxide dismutase (SOD), glutathione peroxidase (GPx), glutathione (GSH) synthesis by glutamate-cysteine ligase modifier subunit (GCLM) and catalytic subunit (GCLC), and heme oxygenase-1 (HO-1) [8]. Previous studies have shown that Nrf2 deficiency led to more severe OA development in mice [9], whereas Nrf2 activation with massive HO-1 expression decreased cartilage degradation [10]. Accordingly, Nrf2 and its downstream antioxidant proteins may be associated with protection against OA progress.

The trace element zinc (Zn) is a component of more than 300 enzymes and an even greater number of other proteins; hence, this element is essential for human health [11]. Zinc is also involved in oxidative stress, immune responses, homeostasis, apoptosis and aging [12]. Prasad [13] reported that zinc is an inhibitor of NADPH oxidase, a co-factor of SOD, and an inducer of metallothionein. Additional studies have demonstrated that zinc was relative to chondrocyte’s growth, such as zinc at low dose (lower than 0.5 μM), could increase the proliferation of cultured chondrocytes by 40–50% [14], and dietary zinc deficiency could inhibit chondrocyte proliferation in the chicken growth plate [15]. Moreover, zinc could block the initiation or propagation of lipid oxidation by preferentially binding to negatively charged phospholipids to prevent the binding of redox-active metals, such as iron and copper [16]. Monosodium iodoacetate (MIA) is an inhibitor of glyceraldehyde-3-phosphate dehydrogenase and is used worldwide to study human OA for causing chondrocyte death. In rodents, intra-articular MIA injection induces articular cartilage loss which is similar to that noted in human OA [17].

Based on these data, we hypothesized that zinc has the potential to protect against OA progress, which may be associated with its antioxidative capacity and/or interference in cytokine expression. Thus, the aim of the present study was to explore the effect of zinc on chondrocyte in the OA progress in vitro using MIA-treated SW1353 cell culture and in vivo using a MIA-induced OA rat model in which the oxidative stress, antioxidative capacity, cytokine expression and the signal-mediated pathway were examined.

## 2. Materials and Methods

### 2.1. Reagents and Antibodies

MIA, zinc sulfate heptahydrate (ZnSO_4_∙7H_2_O) and all other chemicals of analytical grade were purchased from Sigma-Aldrich Co., LLC. (St. Louis, MO, USA). Protein assay reagents were obtained from Bio-Rad Laboratories (Hercules, CA, USA). The following primary antibodies were used in the Western blot analysis. Rabbit anti-HO-1, anti-Nrf2, and anti-p-AKT antibodies and goat anti-actin antibodies were purchased from Santa Cruz Biotechnology Inc. (Santa Cruz, CA, USA). Mouse anti-Zn/Cu-SOD and anti-Mn-SOD antibodies were purchased from BD (BD Biosciences, San Jose, CA, USA). Rabbit anti-glutathione peroxidase 1 was purchased from Abcam plc. (Abcam, Cambridge, UK). Horseradish peroxidase-conjugated anti-mouse, goat, or rabbit IgG antibodies were purchased from Santa Cruz Biotechnology Inc. (Santa Cruz, CA, USA).

### 2.2. Cell Culture

The chondrosarcoma cell line, SW1353, originating from a human cell line, was purchased from the Bioresource Collection and Research Center (BCRC) of the Food Industry Research and Development Institute in Hsinchu, Taiwan. The cells were cultured at 37 °C with Dulbecco’s modified Eagle medium (DMEM) containing 100 units/mL of penicillin, 100 μg/mL streptomycin (Gibco BRL, Grand Island, NY, USA) and 10% fetal bovine serum (FBS) (HyClone, Auckland, NZ, USA) in a 5% CO_2_ incubator. The cells were seeded at 4 × 10^5^ onto dishes in DMEM for 16 h to enable attachment and subsequently treated with 25 μM zinc in the presence or absence of 5 μM MIA for a further indicated period, followed by analysis of the influences.

### 2.3. Cell Viability Assay

SW1353 cells (8 × 10^4^/well) were seeded in 24-well plates and incubated with 0–6 μM MIA or 0–100 μM zinc for 24 h or 48 h. The cells were harvested, and the viable cells were counted using a dye exclusion technique with 0.4% trypan blue (GibcoBRL, Grand Island, NY, USA) in a hemocytometer. All counts were performed in triplicate.

### 2.4. Effects of GSH, SOD, and PI3K Inhibitors on Cell Viability

SW1353 cells (8 × 10^4^/well) were seeded in 24-well plates and pretreated with 10 μM buthionine sulfoximine (BSO), a GSH inhibitor, 0.2 μM diethyldithiocarbamate (DETC), a SOD inhibitor, or 10 μM LY294002 (LY), a PI3K inhibitor, for 30 min. Afterwards, cells were treated with 5 μM MIA and/or 25 μM zinc for 48 h, then cell viability were assayed. All counts were performed in triplicate.

### 2.5. Measurement of ROS

SW1353 cells (4 × 10^5^) were seeded in a 6-cm dish and treated with MIA and/or zinc for 24 h, and intracellular ROS were detected by using 10 μM 2′,7′-dichlorofluorescein diacetate (DCFH-DA) (Molecular Probes, Eugene, OR, USA) for 30 min at 37 °C as described in our previous study [18]. Each experiment was carried out in triplicate.

### 2.6. Measurement of GSH

SW1353 cells (4 × 10^5^) were seeded in a 6-cm dish and treated with MIA and/or zinc for 48 h, and GSH levels were measured using a GSH assay kit (Cayman Chemical, Ann Arbor, MI, USA) according to the manufacturer’s instructions. Each experiment was carried out in triplicate.

### 2.7. Western Blot Analysis

SW1353 cells (4 × 10^5^) were seeded in a 6-cm dish and treated with MIA and/or zinc for 48 h, and then the cell extracts were prepared for Western blot analyses were as previously described [18]. Each experiment was carried out in triplicate. Herein, the proteins were visualized using chemiluminescence detection (PerkinElmer Life Sciences, Inc., Boston, WA, USA). Actin was used as the internal control, each targeted band was calibrated by respective actin. Afterwards, the data of study group were quantitatively analyzed relative to the control group.

### 2.8. Quantitative Real-Time PCR Analysis (qPCR)

SW1353 cells (4 × 10^5^) were seeded in a 6-cm dish and treated with MIA and/or zinc for 24 h, and then the total RNA was extracted by using REzol reagent (Protech, Taipei, Taiwan) according to the manufacturer’s instructions, as previously described [19]. Each experiment was carried out in triplicate. The complementary DNA (cDNA) was synthesized from random primed reverse transcription from 2 μg of total RNA using M-MLV reverse transcriptase (Promega Corporation, Madison, WI, USA) according to the manufacturer’s instructions. Real-time PCR was performed on a MiniOpticon^TM^ Real-Time PCR Detection System (Bio-Rad Laboratories, Hercules, CA, USA) using iQ^TM^ SYBR^®^ Green Supermix (Bio-Rad Laboratories, Hercules, CA, USA) as previously described [20], was used to confirm the results of real-time PCR. The mRNAs encoding GCLC, GCLM, IL-10, and IL-1β were measured using real-time PCR, with RPS18 mRNA as the housekeeping gene. The primers and amplified products of each gene used in the present study are shown in Table 1. The cycle threshold (*C*_t_) value of the target gene was normalized to RPS18. The data were calculated and expressed as 2^−ΔΔ*C*t^ [21] using MJ Opticon Monitor Analysis software version 3.1 (Bio-Rad Laboratories, Hercules, CA, USA).

### 2.9. Animals and Treatments

Male Wistar rats at 4 weeks of age were purchased from BioLASCO Taiwan Co., Ltd. (Charles River Technology, Taipei, Taiwan). Wistar rats at 5 weeks of age (150–170 g) were used. The present study was performed in accordance with the Guide for the Care and Use of Laboratory Animals of the United States National Institutes of Health. The protocol for animal use was reviewed and approved by the Institutional Animal Care and Use Committee (IACUC) of Kaohsiung Medical University (Approval No. 104059; Approval date: 13 August 2015). Sixty male Wistar rats were randomly assigned to six groups containing 10 rats each. The rats were anesthetized using a Tiletamine and Zolazepam mixture (Zoletil 50) (Virbac, Carros, France) and a single intra-articular injection of either sterile saline or MIA (Sigma-Aldrich, St. Louis, MO, USA) at a dose of 3 mg MIA in 20 μL of 0.9% sterile saline was administered through the infrapatellar ligament of the left knee using a 31-gauge needle. Control animals were administered a single intra-articular injection of 20 μL 0.9% sterile saline into the left knee. After the single dose of MIA injection to mimic the OA progress, rats were randomly assigned to three of the six treatment groups, which were one untreated or two zinc supplemented groups. Zinc supplemented groups received water-dissolved zinc of the recommendations of daily dose of 1.6 mg zinc/kg/day [22] or a high dose of 8.0 mg zinc/kg/day by gavage for 2 weeks. All rats were fed standard rodent chow with zinc 70 ppm (Altromin, Lage, Germany). At the end of the experiment period, the rats were sacrificed using CO_2_, and the left leg was removed for histomorphometric analyses. All samples were stored at −80 °C until further analysis.

### 2.10. Histopathology of Joint Tissues: Safranin O and Fast Green Staining

The histopathology of the rat left joints, stained with safranin O and fast green (Sigma-Aldrich, St. Louis, MO, USA), was analyzed as previously described [23]. The histological score of knee joints was assessed by the OARSI cartilage degeneration score [24].

### 2.11. Serum Biomarkers Measurements

The rats (*n* = 10 per group) were sacrificed, and the serum was obtained by centrifuging the blood samples at 3000× *g* for 15 min. The resulting serum samples were divided into aliquots and frozen at −80 °C until further use. There was no repeated freezing and thawing of specimens prior to obtaining measurements. The inflammatory marker IL-1β and anti-inflammatory maker IL-10 were assayed using rat IL-1β and IL-10 ELISA kits (Elisa kit, Antibody-Sunlong Biotech Co., Ltd., Hangzhou, China), respectively. The ECM degrading enzymes MMP-1 and MMP-13 were assayed using rat MMP-1 and MMP-13 ELISA kits, respectively (Elisa kit, Antibody-Sunlong Biotech Co., Ltd., Hangzhou, China). The antioxidant and GSH activities were assayed using an antioxidant assay kit and a GSH assay kit (Cayman Chemical, Ann Arbor, MI, USA), respectively. All samples were examined in triplicate within each assay.

### 2.12. Statistical Analysis

All data are presented as the means ± standard deviation (S.D.). The differences between control and treated groups were analyzed using ANOVA, followed by Fisher’s Exact Test. All statistical analyses were performed using SAS version 6.011 software (SAS Institute Inc., Cary, NC, USA). A *p* value < 0.05 was considered statistically significant.

## 3. Results

### 3.1. Cell Viability

**Figure 1:**
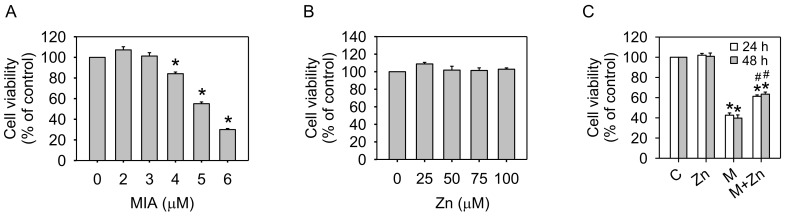
Effects of MIA and/or zinc on the viability of SW1353 cells. SW1353 cells were incubated without/with: (**A**) 0–6 μM MIA for 24 h; (**B**) 0–100 μM zinc for 24 h; or (**C**) 5 μM MIA and 25 μM of zinc for 24 h or 48 h, and subsequently counted using trypan blue exclusion and expressed as a percentage of the control. The results are expressed as the means ± S.D. for three separate experiments, each in triplicate. *: *p* < 0.05 compared to the untreated control group. #: *p* < 0.05 compared to the MIA-treated group.

To obtain an initial insight into the responses of SW1353 cells to MIA treatment, the cells were incubated for 24 h alone or in the presence of 0–6 μM MIA. Cell viability was not affected by 2 or 3 μM MIA but showed dose-dependent cell death at higher MIA concentrations (Figure 1A). Cell viability was approximately 50% after treatment with 5 μM MIA; thus, 5 μM MIA was selected to treat cells, mimicking OA conditions. However, to determine whether zinc treatment affected cell viability, the cells were incubated for 24 h alone or in the presence of 0–100 μM zinc. As shown in Figure 1B, cell viability was not altered by zinc, even at a concentration of 100 μM. Moreover, the addition of 25 μM of zinc to 5 μM MIA-treated cells showed an increase in cell viability (Figure 1C), but higher concentrations were not more effective (data not shown). Therefore, we selected 5 μM MIA and 25 μM zinc for the following cell culture experiments.

### 3.2. Oxidative Stress and Antioxidants

**Figure 2:**
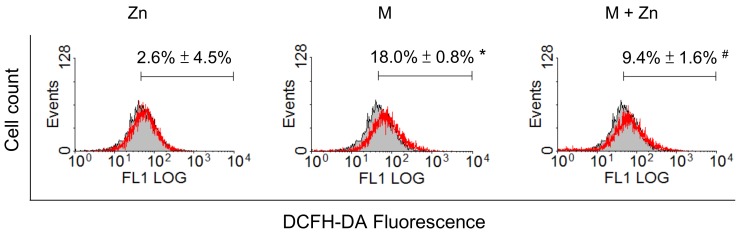
Effects of MIA and/or zinc on oxidative stress in SW1353 cells. SW1353 cells were incubated with 5 μM MIA in the presence or absence of 25 μM zinc for 24 h, and, subsequently, ROS production was measured using 10 μM of DCFH-DA staining, followed by flow cytometry analysis. The gray filled area represents the untreated control, and the red lines represent the treated groups. The results are expressed as the means ± S.D. for three separate experiments, each in triplicate. *: *p* < 0.05 compared to the untreated control group. #: *p* < 0.05 compared to the MIA-treated group.

**Figure 3:**
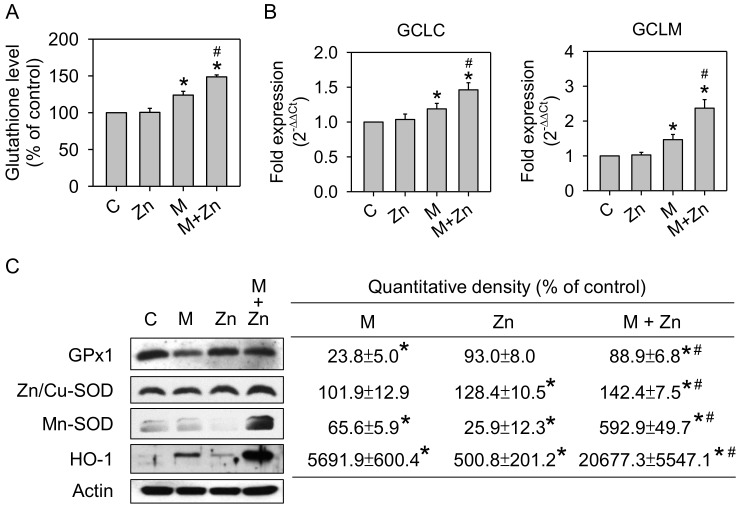

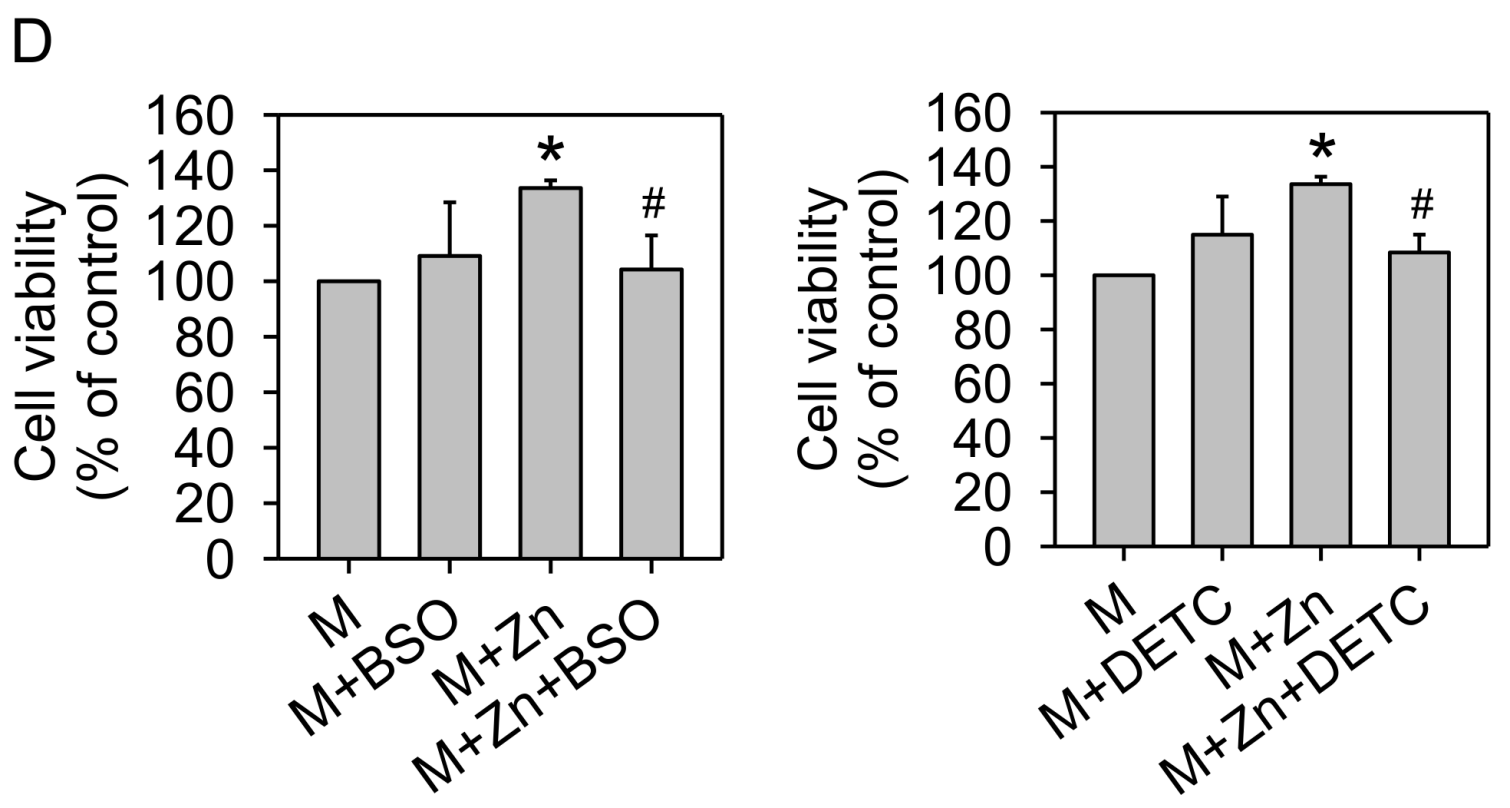
Effects of MIA and/or zinc on the expression of antioxidants. SW1353 cells were left untreated or treated with 25 μM zinc in the presence or absence of 5 μM MIA. (**A**) After 48 h, the GSH levels were assayed. The values were normalized to their protein contents and expressed as a percentage of the control group. (**B**) The mRNA expression of GCLC and GCLM was assayed after 24 h using RT-qPCR and expressed as a fold-change with respect to the untreated control group. (**C**) GPx1, Zn/Cu-SOD, Mn-SOD, and HO-1 expression was detected after 48 h by Western blotting. Actin was used as the internal control. The data in the right panel are expressed as the relative density compared to the untreated cells (control), which was 100%. The results are expressed as the means ± S.D. for three separate experiments, each in triplicate. *: *p* < 0.05 compared to the untreated control group. #: *p* < 0.05 compared to the MIA-treated group. (**D**) The cells were treated with 5 μM MIA in the presence or absence of 25 μM zinc without/with 10 μM BSO or 0.2 μM DETC for 48 h, and, subsequently, the cells were counted using trypan blue exclusion. The data are expressed as a percentage of the MIA-treated group. The results are expressed as the means ± S.D. for three separate experiments, each in triplicate. *: *p* < 0.05 compared to the MIA-treated group. #: *p* < 0.05 compared to the MIA and zinc-treated group.

The results of a previous study indicated that MIA toxicity in chondrocytes reflects MIA-induced oxidative stress [23]. Herein, we examined oxidative stress by evaluating ROS production in SW1353 cells treated with 5 μM MIA in the presence or absence of 25 μM zinc for 24 h. As shown in Figure 2, MIA induced ROS production, while zinc addition would decrease ROS production. Furthermore, we examined whether the levels of antioxidants were changed. The GSH levels were not changed by zinc, but were increased by MIA. Interestingly, the addition of zinc augments the increase of GSH by MIA (Figure 3A). Analysis of the mRNA expression of GCLC and GCLM, key enzymes of GSH synthesis, using real-time PCR showed that the expression of both GCLC and GCLM was increased after MIA treatment, and the addition of zinc augmented this MIA-increased expression (Figure 3B), suggesting that the increase in GCLC and GCLM expression contributes to increased GSH levels. The expression of the antioxidative enzymes GPx1, Zn/Cu-SOD, Mn-SOD, and HO-1 was analyzed using Western blotting, and Figure 3C shows that zinc alone significantly decreased Mn-SOD expression and increased HO-1 expression; MIA decreased GPx1 and Mn-SOD expression but increased HO-1 expression; and the addition of zinc and MIA could massively increase the expression of GPx1, Zn/Cu-SOD, Mn-SOD, and HO-1 as compared with MIA-treated group. These results demonstrate that zinc reduces MIA-induced oxidative stress, reflecting an enhancement of antioxidative enzyme expression. To examine this theory, an inhibitor of GSH or SOD was added to zinc and MIA-treated cells, and, subsequently, cell viability was analyzed. As shown in Figure 3D, the addition of either BSO, a GSH inhibitor, or DETC, a SOD inhibitor, would abate the effects of zinc, indicating that antioxidants are important for the effects of zinc on MIA-treated cells.

### 3.3. Expression of Cytokines and MMPs

**Figure 4:**
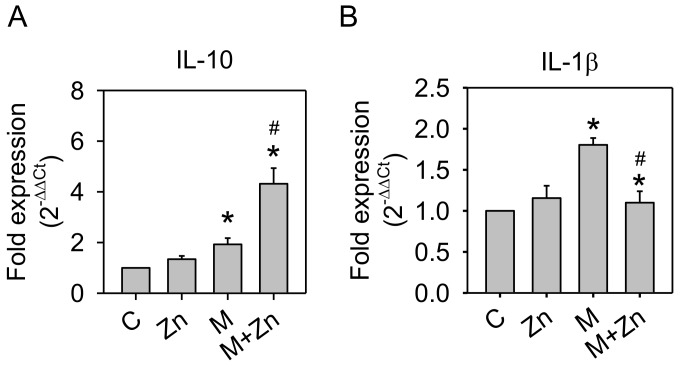

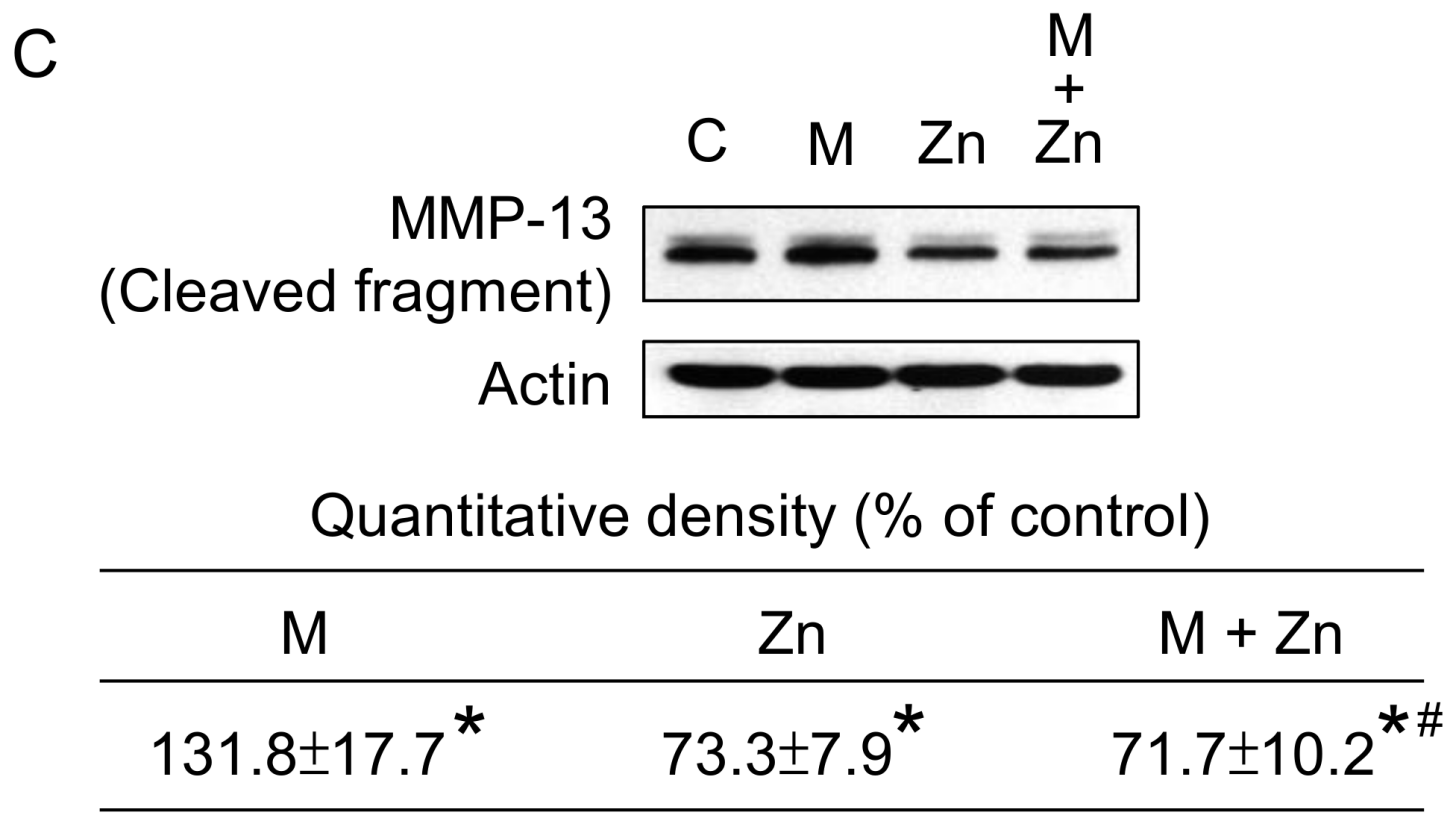
Effects of MIA and/or zinc on IL-10, IL-1β, and MMP-13 expression. SW1353 cells were treated with 5 μM MIA in the presence or absence of 25 μM zinc for the indicated incubation times. (**A**,**B**) The mRNA expression of IL-10 and IL-1β (24 h) was assayed using RT-qPCR and expressed as a fold-change with respect to the untreated control group. (**C**) MMP-13 protein expression (48 h) was determined using Western blotting. Actin was used as the internal control. The results are expressed as the means ± S.D. for three separate experiments, each in triplicate. *: *p* < 0.05 compared to the untreated control group. #: *p* < 0.05 compared to the MIA-treated group.

Fernández et al. [25] reported that the anti-inflammatory cytokine, IL-10, could enhance HO-1 protein expression in human osteoarthritic chondrocytes. In addition, IL-10 exhibits protective effects in the course of OA [26]. We further examined whether the increased HO-1 expression was associated with IL-10 mRNA expression. As shown in Figure 4A, MIA increased IL-10 mRNA expression, and the addition of zinc to MIA-treated cells could augment this increase, similar to the trend of HO-1 changes. Studies also reported that IL-10 blocked pro-inflammatory cytokine secretion and inhibited MMPs production [27,28]. Therefore, we examined the mRNA expression of IL-1β, a pro-inflammatory cytokine, using real-time PCR and characterized active MMP-13 protein expression using Western blotting. Figure 4B shows that IL-1β mRNA expression was increased by MIA, while zinc decreased MIA-induced IL-1β mRNA expression. Figure 4C shows a similar trend of active MMP-13 expression. These results indicate that IL-10 may play an important role in the changes of HO-1, IL-1β, and MMP-13 by MIA in the absence or presence of zinc treatment.

### 3.4. Expression of Phosphorylated-Akt and Nrf2 Expression

**Figure 5:**
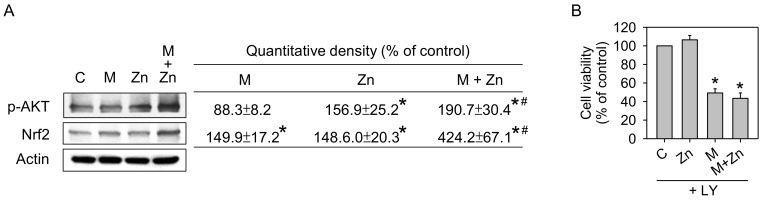
Effects of MIA and/or zinc on the p-Akt/Nrf2 signaling pathway. (**A**) SW1353 cells were left untreated or treated with 25 μM zinc in the presence or absence of 5 μM MIA, and, after 48 h incubation, the proteins levels of p-Akt and Nrf2 were detected using Western blotting. Actin was used as the internal control. The data in the right panel are expressed as the relative density compared to the untreated cells (control), which was 100%. (**B**) SW1353 cells pretreatment with the 10 μM PI3K inhibitor, LY294002 (LY), for 30 min were treated with 5 μM MIA and/or 25 μM zinc for 48 h and subsequently counted using trypan blue exclusion. The data are expressed as a percentage of the control group. The results are expressed as the means ± S.D. for three separate experiments, each in triplicate. *: *p* < 0.05 compared to the untreated control group. #: *p* < 0.05 compared to the MIA-treated group.

Nrf2 is a sensitive mild oxidative stress sensor that regulates antioxidant enzyme expression [29,30]. As shown above, oxidative stress and antioxidative enzyme expression in SW1353 cells were changed by MIA and/or zinc treatment; thus, we determined whether these changes were associated with the expression of Nrf2 or its upstream regulator Akt. Figure 5A shows that zinc alone increased the expression of Nrf2 and active/phosphorylated Akt, while MIA increased Nrf2 expression but did not affect active/phosphorylated Akt expression. The addition of zinc to MIA-treated cells intensively increased both Nrf2 and active/phosphorylated Akt expression, suggesting that Akt/Nrf2 pathways are involved in the effects of zinc and/or MIA on SW1353 cells. To further investigate the role of Akt/Nrf2, LY294002, an inhibitor of the upstream Akt regulator, phosphoinositide 3-kinase (PI3K), was added to zinc and/or MIA-treated cells, and, subsequently, cell viability was assayed. Figure 5B shows that the effect of zinc on the viability of MIA-treated cells disappeared after LY294002 addition. This effect further suggests that PI3K/Akt/Nrf2 pathways participate in the zinc effect on MIA-treated cells.

### 3.5. MIA-Induced OA Progression in Rats, with/without Zinc Supplementation

**Figure 6:**
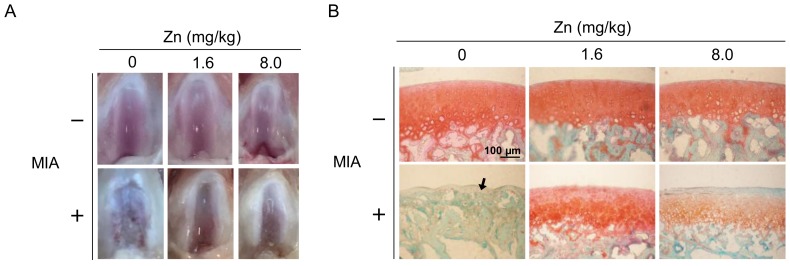

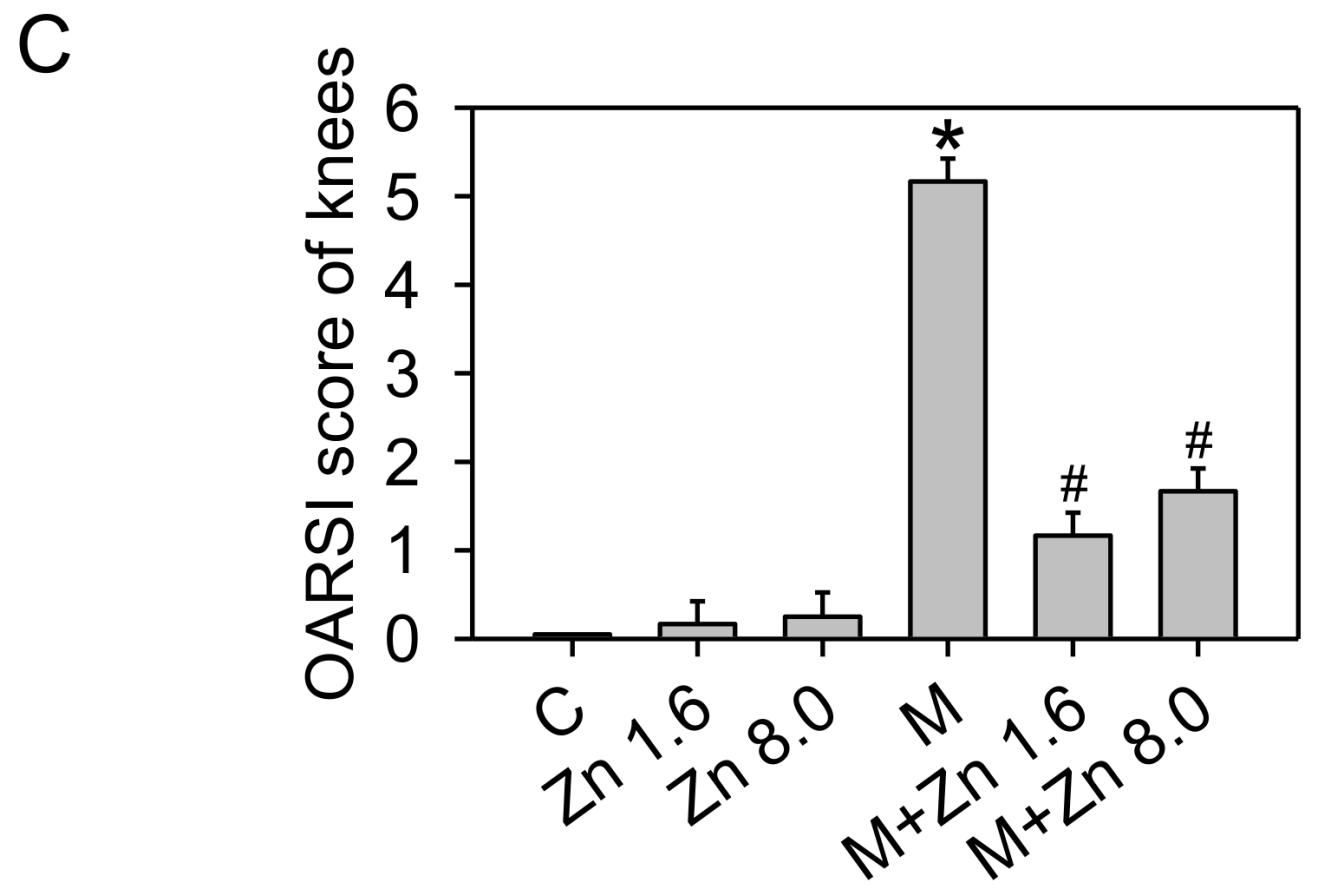
Effects of zinc on the morphology and histology of articular cartilage in an MIA-induced OA rat model. The study schema and experimental timeline of MIA-induced OA in rats and zinc administration are described in the Materials and Methods. (**A**) Images of the articular surfaces of the femoral groove. (**B**) Photomicrographs of histomorphological changes of joint cartilage stained with safranin O/fast green. In these images, red represents proteoglycan. Black arrow indicates the loss of proteoglycan after MIA injection. (*n* = 10 per group). Scale bar: 100 μm. (**C**) OARSI score of each joint. (*n* = 10 per group). *: *p* < 0.05 compared to the untreated control group. #: *p* < 0.05 compared to the MIA-treated group.

**Figure 7:**
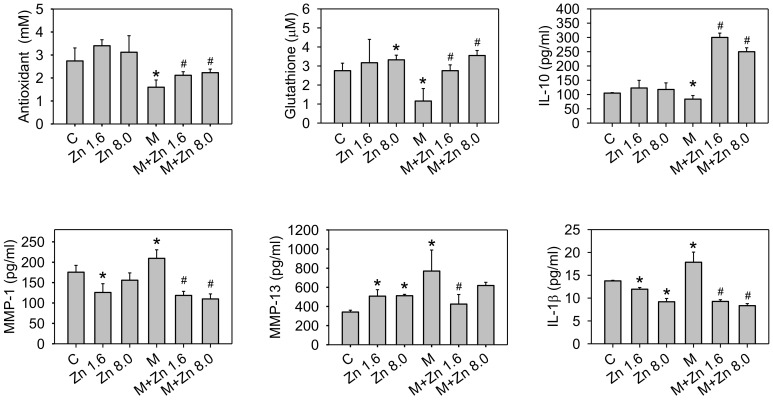
Effects of zinc on the serum levels of antioxidants, MMPs, and cytokines in rat with MIA-induced OA. After treatment as described in the Materials and Methods for two weeks, The antioxidative capacity of the serum as well as the serum levels of GSH, IL-10, MMP-1, MMP-13, and IL-1β in the experimental groups were detected. The results are expressed as the means ± S.D. (*n* = 10 per group). *: *p* < 0.05 compared to the untreated control group. #: *p* < 0.05 compared to the MIA-treated group.

We further examined the effects of zinc on MIA-induced OA in rats to confirm whether the results obtained in cell culture were the same in vivo. After two weeks of experimental treatment, the morphology of the articular cartilage in the zinc supplemented group was same as that of the control group, whereas the MIA-treated group showed severe erosion, synovial hypertrophy and cartilage defect indicating marked arthritic progression (Figure 6A). In addition, zinc supplementation could reduce the arthritic progression in the MIA-treated group. Next, we further examined the histological results of cartilage extracellular matrix proteoglycan (Figure 6B,C). The MIA-treated group had higher Osteoarthritis Research Society International (OARSI) scores and was negative for safranin O staining (red color) and positive for fast green staining indicating no acidic proteoglycan cartilage. The MIA-treated group with zinc supplementation showed smooth joint surfaces, lower OARSI scores, and positive safranin O staining, indicating that proteoglycan was not lost. Notably, the effects of 1.6 mg/kg/day zinc supplementation were not apparently different from those of 8.0 mg/kg/day zinc supplementation. The antioxidative capacity of the serum as well as the serum levels of GSH, IL-10, MMP-1, MMP-13, and IL-1β in treated rats were evaluated, and the results are shown in Figure 7, demonstrating that the zinc supplemented group had higher GSH levels and antioxidative capacity, increased IL-10 and MMP-13 levels, and decreased IL-1β levels compared with the control group. There was no significant difference between 1.6 and 8.0 mg/kg/day of zinc supplementation. The MIA-treated group had massively decreased GSH and antioxidative capacity and lower IL-10 levels but higher MMP-1, MMP-13, and IL-1β levels compared with the control group. Zinc supplementation of MIA-treated rats could inhibit the changes induced by MIA. Notably, IL-10 levels were dramatically increased with zinc supplementation compared to the MIA-treated group, suggesting that IL-10 may play a significant role in the effects of zinc on the MIA-induced OA progress of rats. The in vitro data showed zinc supplementation increased the mRNA expression of GCLC, GCLM, and pro-inflammatory cytokines as well as the protein expressions of antioxidative enzymes, while in vivo rat model zinc supplementation increased serum antioxidative capacity, GSH levels, and IL-10 levels. Taken together, the results of the present study demonstrated that zinc supplementation could prevent MIA-induced OA progress, particularly through the enhancement of antioxidative capacity.

## 4. Discussion

GSH is the first line defense against oxidative damage and can destroy ROS and other free radicals via enzymatic and non-enzymatic mechanisms [31]. The SOD catalyzes oxygen anion to hydrogen peroxide, which is subsequently detoxified by GPx or catalase to water. HO-1 catalyzes the conversion of heme into biliverdin, carbon monoxide and free iron. All these are known as the intracellular defense system against oxidative stress [32,33]. Kloubert and Rink [34] reported that zinc plays an important role in the activation of these defense enzymes. In addition, the production of various pro-inflammatory cytokines, such as IL-1β and IL-6 by macrophages requires zinc signals. Reports further indicated that the enhancing or inhibiting pro-inflammatory cytokines release by zinc depends on zinc dosage used, cell types and experimental condition [35]. Moreover, zinc is recognized as a regulator in signal pathways in immunity and redox metabolism, and is called as zinc signals [36,37].

The results of the present study showed that exposure of 5 μM MIA to SW1353 cells can increase oxidative stress, leading to cell cytotoxicity as previously described [23]. MIA exposure also intensively decreases GPx1 and Mn-SOD expression but increased GSH levels and HO-1 expression. This finding indicates that some antioxidative capacities are induced in defense against MIA-induced oxidative stress. In addition, the expression of IL-10, IL-1β, and MMP-13 is increased. Furthermore, these MIA-induced changes are blocked by supplementation with zinc. Interestingly, the results (Figure 5A) showed that MIA or zinc can stimulate Nrf2 expression, while only zinc stimulates the upstream active regulator, p-Akt. Co-addition of MIA and zinc dramatically increases Nrf2 and p-Akt expression, consistent with the responses of antioxidants, indicating that the p-Akt/Nrf2 pathway is involved in the effects of zinc and/or MIA on SW1353 cells. 

The MIA-induced OA rat model generated results similar to those obtained in cell culture, and 1.6 mg/kg/day zinc supplementation was sufficient to prevent OA progress, while 8.0 mg/kg/day zinc supplementation does not show a better effect. Notably, zinc supplementation to rats without MIA treatment decreased the serum IL-1β levels indicating immunosuppression was induced by the supplemented dosage of either 1.6 mg/kg/day or 8.0 mg/kg/day. In contrast, MIA treatment dramatically increased serum IL-1β levels indicating the immunity and inflammation would be activated. Therefore, we proposed that the zinc supplementation to the MIA-treated rats could lessen inflammation due to its immunosuppression effect. These findings indicate that zinc supplementation inhibits MIA-induced OA progress in vitro and in vivo through changes in antioxidative capacity, pro-inflammatory cytokines and MMPs (decrease protein level of MMP-13 in vitro, and MMP-1 and MMP-13 in vivo). In addition, according to in vitro study, the protective effects exert through the p-Akt/Nrf2 signaling pathway.

The increase in oxidative stress of MIA-treated chondrocytes observed in the present study is consistent with the results of a previous study [23] and other reports [38]. GSH is an important antioxidant that can destroy ROS and other free radicals through enzymatic and non-enzymatic mechanisms [31]. Studies have reported that patients with osteoarthritis have lower GSH levels in erythrocytes [39] and joint fluids [40]. In addition, the levels of GPx, Mn-SOD, and GCLC and GCLM, rate-limiting enzymes for GSH synthesis, were decreased in OA cells [41,42,43,44], in contrast to the results obtained in cell culture showing increased GSH levels after MIA treatment. However, in vivo experiments showed a decrease of GSH levels in the serum of rats in MIA-induced OA progress. We propose that the increase in GSH levels of cells is a necessary response to MIA-induced oxidative stress, since GSH is the first-line defense and may gradually be decreased as the other lines of defense do not work efficiently or decay. Consistently, GPx1 and Mn-SOD expression was decreased in SW1353 cells after MIA treatment. Importantly, this study showed that zinc supplementation to MIA-treated cells can enormously increase the protein expression of GPx1, Zn/Cu-SOD, Mn-SOD, and HO-1, and the mRNA expression of GCLC and GCLM, rate-limiting enzymes for GSH synthesis. Among these factors, Zn/Cu-SOD, GCLC, GCLM, and GPx participate in the antioxidant activity in the cytosol, and Mn-SOD and GPx participate in mitochondrial antioxidant activity [45].

Nrf2, a transcription factor, is a master regulator of antioxidant defense genes which regulate the redox imbalance through the upregulation of antioxidant response element (ARE)-responsive antioxidant enzymes [46]. Reports have shown that Nrf2-knockout mice had more severe cartilage damage in MIA-induced OA models [9], and Nrf2 activation could decrease MIA-induced oxidative stress in chondrocytes [47]. Studies have also reported that the activation of the PI3K-Akt signaling pathway promotes matrix synthesis [48] and enhances the survival of chondrocytes [49]. These results are consistent with reports that the activation of the p-Akt/Nrf2 pathway can reduce damage to chondrocytes through the regulation of downstream effectors, such as antioxidant enzymes.

Bach1^−/−^ mice with deficiencies in the transcriptional repressor of HO-1 showed severe OA-like changes, and these changes could be reduced through the upregulation of HO-1 expression [50]. In addition, HO-1 expression has been associated with the expression of MMPs and pro-inflammatory cytokines in human articular chondrocytes [51]. IL-10 stimulates HO-1 expression in human osteoarthritic chondrocytes [25]. In addition, IL-10 blocks pro-inflammatory cytokine secretion and inhibits MMPs production in chondrocytes [25,26]. The results of the present study provide consistent evidence that zinc supplementation to MIA-treated cells massively increases IL-10 mRNA expression and HO-1 protein expression and decreased IL-1β mRNA expression and MMP-13 protein expression. Moreover, in the MIA-induced OA rat model, zinc supplementation also dramatically increases serum IL-10 levels and decreases MMP and IL-1β levels. These results suggest that IL-10 participates in the effect of zinc on OA progress.

**Figure 8:**
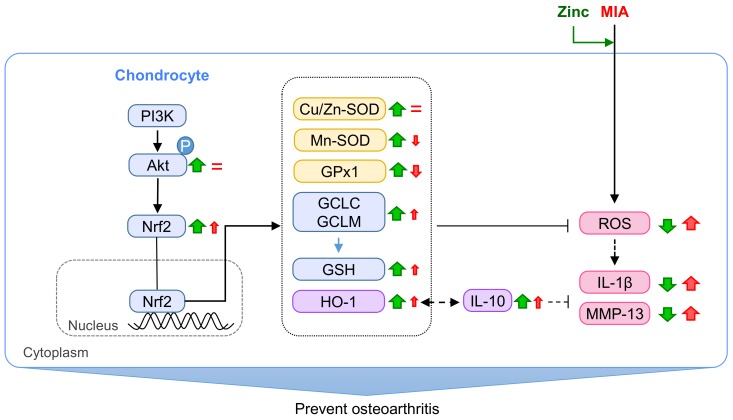
Schematic diagram of zinc effects on MIA-treated chondrocytes. The results of the present study demonstrates that zinc can protect against MIA-increased oxidative stress, pro-inflammatory cytokines (IL-1β), and MMPs through the activation of the Akt/Nrf2 pathway, which upregulates the gene expression of antioxidants, such as Cu/Zn-SOD, Mn-SOD, GPx1, GSH, GCLC and GCLM, and HO-1, leading to the increased antioxidative capacity in defense against MIA-induced oxidative stress. In addition, IL-10 expression is relatively slightly increased by MIA and massively increased after zinc addition, leading to decreased IL-1β and MMPs expression and increased HO-1 expression. Red↑: enhanced by MIA; Red↓: decreased by MIA; Red=: did not change by MIA; Green↑: enhanced by zinc; Green↓: decreased by zinc.

In summary, as shown in Figure 8, the present study demonstrates that in SW1353 cells, MIA exposure induces oxidative stress, decreases the expression of the antioxidative enzymes GPx1 and Mn-SOD, and increases the levels of pro-inflammatory cytokines IL-1β and MMP-13. The addition of zinc to MIA-treated cells activates the PI3K/Akt/Nrf2 pathway to increase antioxidative activity in defense against oxidative stress and decrease IL-1β and MMP-13 levels. Notably, the results observed in vitro were similar to those observed in vivo. These results indicate that zinc can prevent against MIA-induced OA progress.

## 5. Conclusions

In conclusion, the results of the present study demonstrate, both in vitro and in vivo, zinc can prevent against MIA-induced changes in cartilage degradation similar to human OA. It suggests that zinc has the potential to be a preventive supplement for OA in humans.

## Figures and Tables

**Table 1:** Primer sets for qPCR analysis.

Primer Name	NCBI Reference Sequence	Primer Sequence (5′ → 3′)
RPS18	NM_022551.2	F: GAGGATGAGGTGGAACGTGT
		R: TCTTCAGTCGCTCCAGGTCT
GCLC	NM_001498	F: GAGGTCAAACCCAACCCAGT
		R: AAGGTACTGAAGCGAGGGTG
GCLM	XM_005270754.3	F: CTTGGAGCATTTACAGCCTTAC
		R: GGTGGCATCACACAGCAG
IL-10	NM_000572.2	F: GGCTTCCTAACTGCTACAAATAC
		R: AATCCCTCCGAGACACTGG
IL-1β	NM_000576.2	F: TGATGGCTTATTACAGTGGCAATG
		R: GTAGTGGTGGTCGGAGATTCG

RPS18: *Homo sapiens* ribosomal protein S18; GCLC: Glutamate-cysteine ligase catalytic subunit; GCLM: Glutamate-cysteine ligase modifier subunit; IL: Interleukin; F: Forward primer; R: Reverse primer.

## Abbreviations

OA	Osteoarthritis
Zn	Zinc
MIA	Monosodium iodoacetate
p-Akt	Phosphorylated-Akt
ECM	Extracellular matrix
IL	Interleukin
MMP	Matrix metalloproteinase
ROS	Reactive oxygen species
Nrf2	Nuclear factor erythroid 2-related factor
SOD	Superoxide dismutase
GPx	Glutathione peroxidase
GSH	Glutathione
GCLC	Glutamate-cysteine ligase catalytic subunit
GCLM	Glutamate-cysteine ligase modifier subunit
HO-1	Heme oxygenase-1
BSO	Buthionine sulfoximine
DETC	Diethyldithiocarbamate
PI3K	Phosphoinositide 3-kinase
OARSI	Osteoarthritis Research Society International
